# Feasibility, acceptability, and effectiveness of web-based and mobile PTSD Coach: a systematic review and meta-analysis

**DOI:** 10.1080/20008066.2023.2209469

**Published:** 2023-05-25

**Authors:** E. Bröcker, S. Suliman, M. Olff, S. Seedat

**Affiliations:** a Department of Psychiatry, Faculty of Medicine and Health Sciences, Stellenbosch University, Cape Town, South Africa; b Department of Psychiatry, Academic Medical Centre, University of Amsterdam, Amsterdam, the Netherlands

**Keywords:** PTSD Coach, PTSD Coach online, feasiblity, acceptability, effectiveness, Entrenador de TEPT, Entrenador de TEPT en línea, Factibilidad, Aceptabilidad, Eficacia, PTSD教练, PTSD在线教练, 可行性, 可接受性, 有效性

## Abstract

**Background:** Timely and effective interventions can alleviate or prevent the development of clinical symptomatology in trauma-exposed individuals. However, due to limited access to these interventions, and/or stigma around accessing mental health services, there is an unmet need. Internet-and mobile-based interventions may help to address this need.

**Aims:** This review aims to (i) synthesise the evidence on feasibility, acceptability, and effectiveness of the ‘PTSD Coach’ intervention (both web-based and mobile applications) in trauma-exposed individuals; (ii) evaluate the quality of this research; and (iii) identify challenges and recommendations related to ‘PTSD Coach’ intervention delivery.

**Method:** Systematic database searches were conducted (PubMed/MEDLINE, PsycINFO, EMBASE, PLoS, Web of Science, PTSDpubs, Scopus, and clinical trial databases). Review inclusion was based on predefined inclusion criteria, and study quality was assessed with the mixed methods appraisal and the risk-of-bias tools for randomised trials. Where feasible, meta-analytical pooling of intervention effects on posttraumatic stress symptoms (PTSS) was conducted.

**Results:** Seventeen manuscripts reporting on 16 primary studies were included with the majority evaluating a self-managed PTSD Coach mobile application intervention. Most studies were conducted in higher-income countries and females were over-represented. For both platforms, satisfaction and perceived helpfulness were generally high but type of smart device operating system was identified as an influence. The pooled effect size in symptom severity in the intervention group compared to the comparison group was not significant (standardised mean difference =  – 0.19) (95% CI − 0.41 to − 0.03, *p* = .09). Heterogeneity was not significant (*p* = .14; I2 = 40%). No study was excluded based on quality assessment.

**Conclusion:** Findings support the feasibility and acceptability of ‘PTSD Coach’ in trauma-exposed individuals. However, evidence on the effectiveness on PTSS remains limited. More research is still needed in low-middle-income countries, particularly those in which supported ‘PTSD Coach’ interventions are evaluated in larger and more diverse samples.

## HIGHLIGHTS

- Most studies evaluated a self-managed PTSD Coach mobile application intervention in higher-income countries.
- Findings generally support the feasibility and acceptability of both PTSD Coach mobile application and PTSD Coach Online in trauma-exposed individuals but the evidence on the effectiveness on posttraumatic stress symptoms remains limited.
- More research is needed especially in low-middle-income countries where there is often limited access to the needed interventions.

## Abstract (es)

**Antecedentes:** Las intervenciones oportunas y eficaces pueden aliviar o prevenir el desarrollo de sintomatología clínica en personas expuestas a trauma. Sin embargo, debido al acceso limitado a estas intervenciones y/o al estigma que rodea el acceso a los servicios de salud mental, existe una necesidad insatisfecha. Las intervenciones basadas en Internet y en dispositivos móviles pueden ayudar a cubrir esta necesidad.

**Objetivos:** Esta revisión pretende (i) sintetizar las pruebas sobre la viabilidad, aceptabilidad y eficacia de la intervención “Entrenador de TEPT” (PTSD Coach) (tanto basada en la web como en aplicaciones móviles) en individuos expuestos a trauma; (ii) evaluar la calidad de esta investigación; e (iii) identificar los retos y recomendaciones relacionados con la prestación de la intervención “Entrenador de TEPT”.

**Método:** Se realizaron búsquedas sistemáticas en bases de datos (PubMed/MEDLINE, PsycINFO, EMBASE, PLoS, Web of Science, PTSDpubs, Scopus y bases de datos de ensayos clínicos). La inclusión de las revisiones se basó en criterios de inclusión predefinidos, y la calidad de los estudios se evaluó con la valoración de métodos mixtos y las herramientas de riesgo de sesgo para los ensayos aleatorizados. Cuando fue posible, se realizó un agrupamiento meta analítico de los efectos de la intervención sobre los síntomas de estrés postraumático (SPT).

**Resultados:** Se incluyeron 17 manuscritos que informaban sobre 16 estudios primarios, la mayoría de los cuales evaluaban una intervención autogestionada con una aplicación móvil del Entrenador de TEPT. La mayoría de los estudios se realizaron en países de ingresos altos y las mujeres estaban sobrerrepresentadas. Para ambas plataformas, la satisfacción y la utilidad percibida fueron generalmente altas, pero el tipo de sistema operativo del dispositivo inteligente se identificó como una influencia. El tamaño del efecto agrupado en la gravedad de los síntomas en el grupo de intervención comparado con el grupo de comparación no fue significativo (diferencia de medias estandarizada =  – 0,19) (IC del 95%: – 0,41 a – 0,03; *p* = 0,09). La heterogeneidad no fue significativa (*p* = 0,14; I2 = 40%). No se excluyó ningún estudio en función de la evaluación de la calidad.

**Conclusiones:** Los resultados apoyan la viabilidad y aceptabilidad del “Entrenador de TEPT” en personas expuestas a traumas. Sin embargo, las pruebas sobre la eficacia en el TEPT siguen siendo limitadas. Todavía se necesita más investigación en los países de medianos-bajos ingresos, en particular en aquellos en los que las intervenciones apoyadas por el “Entrenador de TEPT” se evalúan en muestras más amplias y diversas.

## Abstract (zh)

**背景：**及时有效的干预措施可以减轻或预防创伤暴露个体临床症状的发展。然而，由于获得这些干预措施的机会有限，和/或获得心理健康服务的耻辱感，存在未满足的需求。基于互联网和移动端的干预措施可能有助于满足这一需求。

**目的：**本综述旨在 (i) 整合“PTSD 教练”干预（基于网络和移动应用程序）在创伤暴露个体中可行性、可接受性和有效性的证据；(ii) 评估这一研究的质量；(iii) 识别“PTSD 教练”干预实施相关的挑战和建议。

**方法：**进行了系统的数据库搜索（PubMed/MEDLINE、PsycINFO、EMBASE、PLoS、Web of Science、PTSDpubs、Scopus 和临床试验数据库）。综述纳入基于预定义的纳入标准，研究质量通过混合方法评估和随机试验的偏倚风险工具进行评估。在可行的情况下，对创伤后应激症状 (PTSS) 的干预效果进行元分析汇总。

**结果：**报告 16 项主要研究的 17 篇手稿被纳入其中，其中大多数评估了自评式 PTSD 教练移动应用程序干预。大多数研究是在高收入国家进行，女性人数更多。对于这两个平台，满意度和感知有用性通常都很高，但智能设备操作系统的类型被认为是一个影响因素。与对照组相比，干预组症状严重程度的合并效应量大小不显著（标准化平均差异 =  – 0.19）（95% CI – 0.41 至 – 0.03，*p* = .09）。异质性不显著（*P* = .14；I2 = 40%）。没有研究因质量评估被排除。

**结论：**研究结果支持“PTSD教练”在创伤暴露个体中的可行性和可接受性。然而，关于 PTSS 有效性的证据仍然有限。仍需要在中低收入国家进行更多研究，尤其是那些在更大、更多样化的样本中评估支持性的“PTSD 教练”干预措施的国家。

## 1. Introduction

Trauma exposure (TE) is a risk factor for the development of psychological distress and, in some individuals, the development of trauma – and stressor-related, anxiety and depressive disorders (Adams et al., 2018; Levin-Rector et al., 2018; Suliman et al., 2014). The Diagnostic and Statistical Manual 5th edition (DSM-5) defines TE as experiencing, witnessing, or being confronted with actual or threatened death, serious injury, and/or sexual violence (American Psychiatric Association, 2013). Posttraumatic stress symptoms (PTSS) that can develop in response to a TE can be characterised in four clusters: (i) re-experiencing or intrusion, (ii) avoidance, (iii) negative cognition and mood, and (iv) arousal and reactivity (Weathers et al., 2013). Some trauma-exposed individuals may develop clinically significant PTSS meeting the threshold for posttraumatic stress disorder (PTSD) diagnosis, while others may present with sub-threshold PTSS (Cukor et al., 2010). Regardless, coping mechanisms are often overwhelmed and daily life activities can be negatively affected requiring prompt intervention (Morabito et al., 2020; Skeffington et al., 2020).

Timely and effective interventions can either alleviate or prevent the development of clinical symptomatology (Colizzi et al., 2020). However, many trauma-exposed individuals do not receive timely and effective assistance due to limited access and/or stigma around accessing mental health services (Becker & Kleinman, 2013; Semo & Mogga Frissa, 2020). In response, internet-and mobile-based interventions are increasingly used either as first-line treatment (e.g. whilst on waiting lists for professional mental health care) or as augmentation to mainstream therapeutic modalities (Owen et al., 2015; Price et al., 2014).

In 2011, a freely available self-managed mobile application-based intervention called PTSD Coach was introduced by the United States Departments of Veterans Affairs and Defense (VA/DoD) (Owen et al., 2015). The trauma-focused PTSD Coach mobile application aims to provide trauma-exposed individuals with psycho-education on trauma and PTSD, assist with managing and monitoring posttraumatic stress symptoms (PTSS), and encourage seeking treatment if so indicated. These aims are addressed through the four core sections of the mobile application: (1) ‘Learn’, (2) ‘Track progress’, (3) ‘Manage symptoms’, and (4) ‘Get support.’ Whereas ‘Learn’ provides the mentioned psycho-education, ‘Track progress’ refers to the brief assessments designed to assist individuals with symptom monitoring (e.g. rating anxiety level before and after accessing a specific symptom management tool). These assessments accompany the 21 evidenced-based tools under ‘Manage symptoms.’ Informed by both mindfulness and cognitive behavioural therapy principles, these easily accessible evidenced-based tools were designed to address eight core PTSD symptoms, namely, being reminded of the trauma, avoiding triggers, being disconnected from people, disconnected from reality, sad/hopeless, worried/anxious, angry, and unable to sleep. The mobile application's ‘Get support’ section includes a feature to customise contact details of applicable support services and safety plans when indicated. PTSD Coach does not require data post download nor collects identifiable data during usage and can be used independently as a self-management tool or as augmentative to other psychological support treatments (Kuhn et al., 2018).

A VA/DoD review summarised the primary research data of eight of their 20 available mobile application interventions, of which the well-documented PTSD Coach was one (Gould et al., 2019). The reviewers concluded that comparatively more research has been conducted on the ‘PTSD Coach’ and the ‘Virtual Hope Box’ mobile applications, and further evidence regarding effectiveness was needed.

Since its release, the PTSD Coach mobile application has been translated and culturally adapted in six countries (Australia, Canada, The Netherlands, Germany, Sweden, and Denmark) (Kuhn et al., 2018). At the time of the review by Kuhn et al. (2018), no systematic evaluation of PTSD Coach Australia, PTSD Coach Canada, and PTSD Coach Denmark mobile applications were conducted, and primary research had been conducted on SUPPORT Coach (The Netherlands) and PTSD Coach Sweden mobile applications.

Cumulatively, the developing research is promising as the results suggest high user satisfaction and acceptability, as well as self-reported PTSS improvement. Kuhn et al. (2018) concluded that even though the PTSD Coach mobile application is progressively expanding its reach, most of the research is conducted in higher-income settings with known higher smartphone ownership and higher mental health resource access.

In addition to the VA/DoD's research advances of the PTSD Coach mobile application (hereafter referred to as PTSD Coach MA) and general internet-based intervention development, a web-based ‘PTSD Coach Online (PCO)’ platform was developed and released in 2013 (National Center for PTSD, 2017). These two platforms are similar in that both provide easily accessible support based on user needs, are informed by mindfulness and cognitive behavioural therapy principles, and can be used as a self-management tool or augmentative to other support. However, the web-based platform includes additional psycho-educational videos with instructions for completing various computer-based interactive activities and requires an active internet connection.

### 1.1. Review aims

This mixed methods review of controlled and uncontrolled clinical studies on ‘PTSD Coach’ (web-based and mobile application platform) aims to (i) synthesise the evidence on feasibility, acceptability, and effectiveness in trauma-exposed individuals; (ii) assess the quality of this research; and (iii) identify challenges and recommendations related to PTSD Coach intervention delivery. For this review, feasibility and acceptability were determined by considering attrition rates, PTSD Coach MA/PCO usage, smart device ownership, and feedback provided by trauma-exposed study participants on perceived satisfaction/benefits and barriers/concerns. Effectiveness was determined by considering a reduction in PTSS evaluated with validated subjective or diagnostic measures (i.e. PTSD Checklist/Clinician-administered PTSD Scale).

## 2. Methods

### 2.1. Registration and protocol

This systematic review was registered with the International Prospective Register of Systematic Reviews (PROSPERO) database (CRD42022331731). It conforms with both the Cochrane Collaboration guidelines and the Preferred Reporting Items for Systematic Reviews and Meta-analyses (PRISMA) reporting standards (Liberati et al., 2009).

### 2.2. Eligibility criteria

Only peer-reviewed primary research published in English was considered for the review. Specifically, this review considered quantitative, qualitative, or mixed methods primary research reporting on the feasibility, acceptability, and effectiveness of either ‘PTSD Coach’ platforms. We excluded secondary research papers such as narrative, scoping, and systematic reviews. We included studies utilising any of the two ‘PTSD Coach’ platforms as an intervention for trauma-exposed individuals (with and without a PTSD diagnosis) of all ages in any setting. A study was eligible for inclusion if feasibility, acceptability, or effectiveness was assessed (i.e. a minimum of any one of these domains was required for inclusion), and included methods (i.e. assessments/tools) of assessing the index domains). As this was a mixed methods systematic review, studies were included irrespective of the presence of a control group. Prior to the qualitative and quantitative (meta-analyses) synthesis, the primary reviewers independently conducted standardised searches, systematic review inclusion screening, methodological quality and risk of bias assessments, and data extraction (Moher et al., 2001; Viswanathan et al., 2018). The reviewers met to compare their independent findings and minor disagreements were resolved through discussion. An eligibility form was used to capture reasons for exclusion/inclusion (Additional File 1: Eligibility form).

### 2.3. Literature search

We conducted a systematic search between 19 and 26 July 2022 of the following electronic databases: PubMed/MEDLINE, PsycINFO, EMBASE, PLoS, Web of Science, PTSDpubs, and Scopus. Clinical trial registries (ClinicalTrials.gov; International Clinical Trials Registry Platform; Pan-African Clinical Trials Registry; International Standard Randomised Controlled Trial Number) were searched for additional relevant studies not identified by the initial database searches. No limitations on the date of publication were applied. References within the bibliographies of identified studies were manually searched for additional relevant studies.

We were unable to find primary research studies on the following three PTSD Coach MA versions: PTSD Coach Canada, CoachPTBS Germany, and PTSD Coach Denmark, which were included in a previous PTSD Coach MA review (Kuhn et al., 2018). Communication with the corresponding author of this review confirmed the accuracy of our search results.

### 2.4. Search strategy and study selection

**Figure 1.**
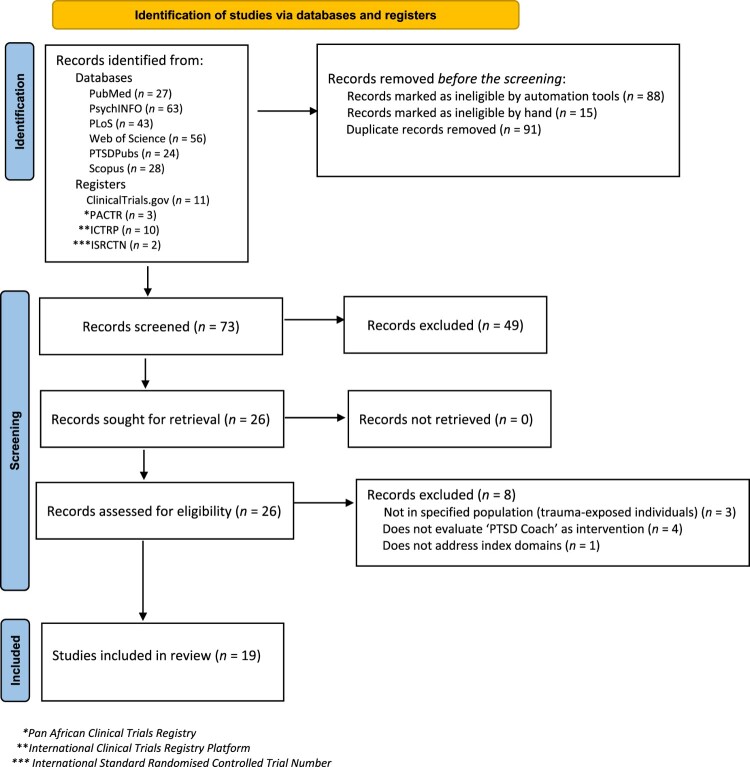
Preferred reporting items for systematic reviews and meta-analysis (PRISMA) flow chart of included studies. From: Page et al. (2021).

The primary search was formulated and conducted in MEDLINE (PubMed) and translated to the other databases. The primary search included the following keywords (and MeSH terms): (‘PTSD Coach’ OR ‘PTSD Coach Online’) AND (stress OR trauma OR mental OR emotion). An information specialist (librarian) assisted with the identification of the correct search strategy and search terms. Figure 1 depicts the PRISMA Flow Diagram (McKenzie et al., 2021). The search identified (*N* = 267) records. We first applied non-primary research filters (i.e. reviews, newsletters, web articles, newsletter articles, reports, web pages, and guidelines) and removed 88 records. Thereafter, an additional 15 records were removed manually as they were identified as reviews or study protocols. After duplicates were removed (*n *= 91), 73 records were exported to Mendeley (reference management software) which was used to save and manage searches (Mendeley, 2009).

Further screening of titles and abstracts based on eligibility criteria (letter to the editor, not PTSD Coach-related primary research, systematic reviews, guidelines) led to the removal of 47 records, resulting in 26 full-text records remaining. An additional eight records were removed resulting in 17 records included in the review. Of the eight excluded records three were not conducted in trauma-exposed individuals; ‘PTSD Coach’ was not evaluated as an intervention (*n* = 4), and one was a review that included the primary studies already included.

### 2.5. Data extraction

The data extraction form used in the review can be accessed in Additional File 2: Data extraction form. The first reviewer extracted the relevant data from the included studies, while the second reviewer corroborated the extracted data. Extracted data included study characteristics; trauma-related data; study methodology; and outcome data. The main outcomes for which the data were sought included: (i) feasibility; (ii) acceptability; and (iii) effectiveness of the PTSD Coach MA and PCO on PTSS, respectively.

### 2.6. Quality assessment

**Figure 2.**
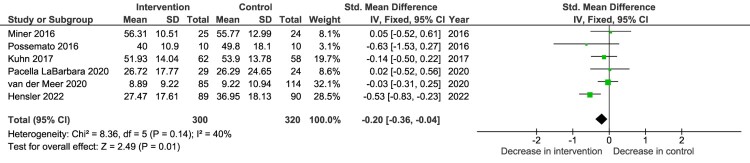
Intervention effects on changes in PTSS severity post-intervention – comparison intervention versus control group.

Reviewers performed quality assessments independently on all manuscripts and resolved disagreements through discussion. The mixed methods appraisal tool (MMAT) was applied to all manuscripts (Hong et al., 2019). The MMAT is divided into five categories that evaluate the methodological quality of qualitative, quantitative (RCTs, non-RCTs, and descriptive), as well as mixed methods studies. Additionally, the risk-of-bias tool for randomised trials (ROB-2) was applied to the RCT data (Sterne et al., 2019). The ROB-2 evaluates the risk of bias due to (i) the randomisation process, (ii) deviations from the intervention, (iii) missing outcome data, (iv) measurement of the outcome, and (v) selection of the reported result. As depicted in Figure 2, the Robvis tool was used to create a traffic light plot to visualise the Rob 2 assessment results (McGuinness & Higgins, 2021).

### 2.7. Synthesis

We provide a general description of eligible manuscripts and participants. Thereafter, we describe the interventions used in each study, including the relevant outcome data. We then summarise findings based on the feasibility, acceptability, and effectiveness of the intervention on PTSS. To synthesise data from RCTs on the effectiveness on PTSS changes, we conducted a meta-analysis to calculate the pooled effect size based on primary outcome data (i.e. PTSD Checklist – PCL) using Review Manager (RevMan) 5.4.1 (Higgins et al., 2022). Means and standard deviations were used for the continuous outcomes. A random-effects model was used to estimate summary effect sizes (ES) with related confidence intervals (CI of 95%). The I^2^ statistic was used to evaluate the heterogeneity of the included studies (Higgins et al., 2022). Lastly, we report the results of the quality assessments.

## 3. Results

### 3.1. Eligible papers

Seventeen manuscripts met the inclusion criteria. Most were published between 2020–2022 (*n* = 8), with five published between 2017–2019, and four published between 2014–2016. Most of the studies were RCTs (*n* = 7), followed by mixed design (*n *= 6), pre-post design (*n *= 2), observational (*n *= 1), and qualitative design (*n *= 1) (Table 1). The 17 manuscripts included 16 primary studies conducted with one manuscript (Bröcker et al., 2022) reporting on two primary studies. Two articles largely report qualitative data from two primary studies (Ellis et al., 2022; Possemato et al., 2017). Most primary studies (*n* = 7) were conducted in the United States of America (Hiratsuka et al., 2019; Kuhn et al., 2014, 2017; Miner et al., 2016; Pacella-LaBarbara et al., 2020; Possemato et al., 2016; Tiet et al., 2019). Two were conducted in Sweden (Cernvall et al., 2018; Hensler et al., 2022), two in South Africa (Bröcker et al., 2022), and two were multi-country (Hallenbeck et al., 2022; Owen et al., 2015). One each was conducted in Australia (Shakespeare-Finch et al., 2020; van der Meer et al., 2020), and Egypt (Miller-Graff et al., 2021).

**Table 1.** Study and baseline sample characteristics.

Study ID (Country)	Design and data collection	Baseline sample size	Sex	Age in yearsMean/range (SD)	Ethnicity	Smart device ownership
Kuhn 2014 (United States of America)	Mixed Pre-post FGD	Total = 45 Pre-post = 45 FGD = 7	M = 34 F = 11	42.25 (14.03)	Caucasian = 46% Black/African = 17.8% Mixed race = 6.7% Other = 29.5%	Own = 44.4%
Owen 2015 (Multi-country)	Mixed App analytics Written reviews	Total = 153 991 Downloads = 153,834 Written reviews = 156	NS	NS	NS	NS
Miner 2016 (United States of America)	Quanti. RCT Pilot Quanti. survey	Total = 49 Int. = 25; Waitlist control = 24 Survey = 38	M = 9 F = 40	45.7 (13.9)	Caucasian = 57.1% Black/African = 8.2% Mixed race = 14.3% Other = 6.1% Missing = 14.3%	Own = 73.5% Provided = 26.5%
Possemato 2016 (United States of America)	Quanti RCT Pilot	Total = 20 Counsellor-supported Int. = 10; Self-managed Int. = 10	M = 19 F = 1	42 (12)	Caucasian = 65% NS = 35%	NS
Possemato 2017 (United States of America)	Mixed Quanti. survey Quali. interviews	Total = 9 Survey = 9 Interviews = 9	NS	NS	NS	NS
Kuhn 2017 (United States of America)	Quanti. RCT	Total = 120 Int. = 62; Waitlist = 58	M = 37 F = 83	39	Caucasian = 66.6% Black/African = 15.8% Other = 30%^1^	Own = 100% (Inclusion criteria)
Cernvall 2018 (Sweden)	Mixed Pre-post Quanti. survey Quali. interview	Total = 11 Pre = 11; Post = 11 Quanti. Survey = 11 Quali. interview = 9	M = 3 F = 8	23 – 55y (38.6)	NS	Own = 100% (Inclusion criteria)
Hiratsuka 2019 (United States of America)	Quanti Pre-post	Total = 24 Pre = 24; Post = 22	M = 7 F = 17	49 (14)	American Indian/Alaska Native = 100%	Own = 100% (Inclusion criteria)
Tiet 2019 (United States of America)	Quanti Pre-post	Total = 29 Pre = 29; Post = 29	M = 28 F = 1	32-72y Median = 61	Caucasian = 48.27% Black/African = 17.24% Mixed race = 3.45% Other = 27.59% Declined = 3.45%	Own = 100% (Inclusion criteria)
Pacella-LaBarbara 2020 (United States of America)	Quanti. RCT	Total = 64 Int. = 33; TAU = 31	M = 24 F = 40	37.0 (12.71) 18 - 65years	Caucasian = 44% Black/African = 42% Mixed race = 6% Other = 8% (5)	Own = 100% (Inclusion criteria) Smartphone or inability to download mobile applications was not a barrier for enrolment.
Shakespear-Finch 2020 (Australia)	Mixed Quanti. survey FGD? Quali. interviews	Total = 53 Quanti. survey = 53 FGD = 29 Quali. interview = 24	M = 28 F = 25	35 – 79years (12.4) Median = 62	NS	Own = 100% (Inclusion criteria) Required remote participation from any location with internet access/cellular service.
van der Meer 2020 (the Netherlands)	Quanti. RCT	Total = 259 Int. = 124; Control = 135	M = 122 F = 137	43.4 (9.7)	NS^2^	Own = 100% (Inclusion criteria)
Hensler 2022 (Sweden)	Quanti. RCT	Total = 179 Int. = 89; Waitlist = 90	Other = 15^3^ F = 164	42.78 (10.90)	NS	Own = 100% (Inclusion criteria)
Miller-Graff 2021 (Egypt)	Quanti. RCT	Total = 87 Int. = 41; Waitlist = 46	NS = 25 F = 62	28.65 (7.37) 20 – 54years	Egyptian = 100%	Own = 100% (Inclusion criteria)
Hallenbeck 2022 (Multi-country)	Observational	Total = 207 001 (downloads)	NS	NS	NS	NS
Ellis 2022 (Egypt)	Quali. interview	Total = 15	M = 4 F = 11	NS	NS	NS
Bröcker 2022a (South Africa)	Mixed RCT Quali. feedback and fieldnotes	Total = 10 Counsellor-supported Int = 5; Enhanced TAU = 5 Quali. feedback and fieldnotes = 10	M = 1 F = 9	24–64 years (M = 46.3; SD = 13.82)	Black/African = 10% Mixed race = 90%	Own = 100% (Inclusion criteria)
Bröcker 2022b (South Africa)	Mixed RCT Quali. Feedback and fieldnotes	Total = 10 Counsellor-supported Int = 5; Self-managed Int = 5 Quali. feedback and fieldnotes = 10	M = 1 F = 9	27–54 years (M = 39.4; SD = 8.14)	Caucasian = 50% Black/African = 20% Mixed race = 20% Declined = 10%	Own = 100% (Inclusion criteria)

Notes*:* ^1^Queried with corresponding author rationale for reported ethnicity numbers resulting in *N *= 135 rather than *N *= 120, participants could self-identify with more than one category, which they did; ^2^Dutch citizen = 96.9% and non-Dutch citizen = 3.1%; ^3^Men, other, or preferred not to answer; a = Pilot 1; b = Pilot 2; F = Female; FGD = Focus Group Discussion; Int. = Intervention; M = Male; NS = Not Specified; PTSD = Posttraumatic Stress Disorder; Quali. = Qualitative; Quanti. = Quantitative; RCT = randomised controlled trials; TAU = treatment as usual.

#### 3.1.1. Descriptive data

Table 1 presents the characteristics of included studies. All studies were conducted in adults. Of the 16 primary studies, most (*n* = 11; 68.75%%) were conducted in high-income countries (HICs) (Cernvall et al., 2018; Hensler et al., 2022; Hiratsuka et al., 2019; Kuhn et al., 2014, 2017; Miner et al., 2016; Pacella-LaBarbara et al., 2020; Possemato et al., 2016; Shakespeare-Finch et al., 2020; The World Bank Group, 2022; Tiet et al., 2019; van der Meer et al., 2020). Three studies (18.75%) were conducted in low-middle-income countries (LMICs) (Bröcker et al., 2022; Miller-Graff et al., 2021), with the remaining two (12.5%) characterised as multi-country (Hallenbeck et al., 2022; Owen et al., 2015). The baseline sample size ranged from 153 991–207 001 for the multi-country studies, and 10–259 for the single-country studies (Bröcker et al., 2022; Cernvall et al., 2018; Hensler et al., 2022; Hiratsuka et al., 2019; Kuhn et al., 2014, 2017; Miller-Graff et al., 2021; Miner et al., 2016; Pacella-LaBarbara et al., 2020; Possemato et al., 2016; Shakespeare-Finch et al., 2020; Tiet et al., 2019; van der Meer et al., 2020).

Females were over-represented (607 / 960; 63.23%) based on the 14 studies that reported on sex (Bröcker et al., 2022; Cernvall et al., 2018; Hensler et al., 2022; Hiratsuka et al., 2019; Kuhn et al., 2014, 2017; Miller-Graff et al., 2021; Miner et al., 2016; Pacella-LaBarbara et al., 2020; Possemato et al., 2016; Shakespeare-Finch et al., 2020; Tiet et al., 2019; van der Meer et al., 2020). In the primary studies that specified ethnicity (*n *= 11), most participants self-identified as Caucasian (189/458; 41.27%) (Bröcker et al., 2022; Hiratsuka et al., 2019; Kuhn et al., 2014, 2017; Miller-Graff et al., 2021; Miner et al., 2016; Pacella-LaBarbara et al., 2020; Possemato et al., 2016; Tiet et al., 2019).

#### 3.1.2. Intervention data

Table 2 summarises the intervention characteristics and outcomes of the included primary studies. Eight of the primary studies (50%) were conducted in community samples (American, South African, Swedish, and Egyptian) followed by four (25%) in veteran samples. The remainder were conducted in the general population (*n* = 2; 12.5%), among healthcare professionals (*n* = 1; 6.25%), and motor vehicle accident emergency care patients (*n* = 1; 6.25%).

**Table 2.** Intervention description and outcome data.

Study First author and year	Intervention						Outcome			
							Feasibility	Acceptability		Effectiveness
	Population	Inclusion criteria *Exclusion criteria*	PTSD Coach Platform	Duration (support)	Follow-up	Outcome variable	Attrition *Usage*	Benefits/Satisfaction	Concerns/Barriers	Change in PTSS (Validated self-report or diagnostic measures)
Khun 2014	Outpatient veterans	Veterans attending Veteran Affairs (VA) PTSD residential treatment programme.	App^1^	3-day app use. (Self-managed)	Post 3-day app use.	15-item self-report PTSD Coach survey (Cronbach's *α* = 0.96) to assess user satisfaction and perceived helpfulness.	0% *NS*	Perceived as helpful (irrespective of smart device ownership). Smartphone owners were significantly more satisfied (*M* = 3.20, *SD* 0.70) than non-owners (*M* = 2.56, *SD* = 1.08). Age was not significantly correlated with overall satisfaction (*r* = −0.18, *p* = .24).	Lack of customisation of exercises and direct access to specific tools deemed problematic.	NS
Owen 2015	General population	iOS / Android App users.	App^1^	Variable and not set. (Self-managed)	1-year observation period.	Mobile analytics. Written Reviews.	153,834 downloads in 86 countries. iOS users = maintain both a higher number and proportion of users across time. 52.1% = used MA at least once post download. 41.6%. = used MA 1-month post download. 28.6% = used MA 3 months post download. 19.4%. = used MA 6 months post download. 10.6% = used MA 1-year post download.	50.9% = of iOS users perceived MA as helpful. 20.4% = Android users perceived MA as helpful.	10% = reported disliking the MA. 33.3% = reported technical difficulties (Android users = relatively higher prevalence than iOS users). 15% offered suggestions for MA improvement (i.e. enable social networking among users, create device-specific versions, provide a journalling feature, and create an integrative feature for use with professional support).	NS
Miner 2016	Community	≥18 years. PTSD Checklist – Civilian version (PCL-C) ≥ 25. Fluent in English. Active e-mail address. *Not in treatment for PTSD.*	App^2^	4 weeks (Self-managed)	Post-intervention at 4 and 8 weeks.	PCL-C	4 weeks = 10% 8 weeks = 18% *4 weeks = used by all participants. Int. group weekly usage = between 1–3 times and 4–6 times (M = 2.65; SD = 1.03).*	± 50% = reported MA is useful to manage symptoms. Learn and Manage Symptoms sections of MA = found moderately helpful. 77% = found self-management of symptoms was most useful.	13.2% = found the MA not useful. Non-smart device owners did not benefit to the same extent as smartphone owners.	Non sig. between conditions effect size estimates (Cohen's *d* = −0.25 to −0.33) for the PCL-C (ITT) from baseline to post-intervention.^3^ Sig. decrease (Cohen's *d* *=* .59; *p = *.05) in total PCL-C scores between baseline (*M* = 63.00; *SD* = 11.28) and post-intervention (*M* = 56.31; *SD* = 10.51).
Possemato 2016 Possemato 2017	Primary care patients (veterans)	Enrolled in VA primary care. Military-related trauma exposure (TE). PCL-5 = ≥ 40. Participants in the CS-PTSD Coach arm of the above study.	App^2^	8 weeks (Self-managed and clinician-supported)	Post-intervention at 8, 12, and 16 weeks.^4^	PCL-S Client Satisfaction Questionnaire. Qualitative feedback	8 weeks = 10% *CS-PTSD Coach participants = MA usage was high over the 8 weeks of active study treatment.*	Clinician support increased satisfaction and engagement with MA. 100% (of qualitative interviewees) = reported good to excellent satisfaction with clinician-supported CS-PTSD Coach intervention. 78% of qualitative interviewees = indicated that CS-PTSD Coach met all or most of their needs.	NS	Non sig. between conditions effect size estimates (Cohen's *d* = -.54) for the PCL-S (ITT). CS-PTSD: Sig. change in total PCL-S scores from baseline (*M* = 51.0; *SD* = 7.7) and post-intervention (*M* = 40.0; *SD* = 10.9). SM-PTSD: Sig. change in PCL-S scores from baseline (*M* = 56.0; *SD* = 15.3) and post-intervention (Mean = 49.8; *SD* = 18.1).
Kuhn 2017	Community	≥18 years Smartphone owners with the ability to download App. TE > 1 month PCL-C ≥ 35 *Not currently in PTSD treatment*	App^2^	12 weeks (Self-managed)	Post-intervention at 12 and 24 weeks.	PCL – C 9 – Item self-report PTSD symptom coping self-efficacy scale (Cronbach's α = 0.75) to assess confidence in managing PTSD symptoms and reaching out for support on a scale from 0 (c*annot do at all*) to 100 (*highly certain can do*).	12 weeks = 14% 24 weeks = 5% *Self-reported MA average days use among completers (M = 2.27; SD = 1.76).*	NS Non sig. change in PTSD symptoms coping SE (Cohen's *d* = .25)	*NS*	Non sig. between conditions effect size estimates (Cohen's *d* = -.41) for the PCL-C (ITT). Int. Group: Sig. change (Cohen's *d = *.41; *p* = .035) in total PCL-C scores from baseline (*M* = 63.19; *SD* = 11.78) and post-intervention (*M* = 51.93; *SD* = 14.04).
Cernvall 2018	Community	≥18 years Swedish speaker. Smartphone access with connectivity Android/iOS App store. TE during the past 5 years. *No severe psychiatric co-morbidity (i.e. psychosis, severe depression, mania, ongoing substance abuse and suicide risk).*	App^5^	4 weeks (Self-managed)	Post-intervention 4-weeks.	PCL-5 PTSD Coach survey. Semi-structured telephonic interview.	0% *NS*	Participants were slightly to moderately satisfied (0.91 and 1.91) with MA. Participants found learning about PTSD, breathing exercises and monitoring of symptoms helpful.	Some participants used MA less than intended due to differences in expectation, eye problems, and perceived difficult MA structure.	Non sig. decrease (Cohen's *d = *.16) in total PCL-5 scores between baseline (*M* = 36.09; *SD* = 14.33) and post-intervention (*M* = 31.68; *SD* = 15.91).
Hiratsuka 2019	American Indian/ Alaskan Native (AI/AN) community	≥18 years. AI/AN heritage. Positive screen (≥3/4) on the Primary Care PTSD (PC-PTSD) screener. ≥1 chronic condition diagnosis (i.e. heart disease, stroke, diabetes, kidney disease, arthritis, osteoporosis, cancer, asthma, depression, and chronic obstructive pulmonary disease). ≥2 primary care clinic visits in the past 12 months. Cellular service for 3 months post enrolment. Access to phone with text-message ability. Access to computer with an internet connection.	PCO^6^	12 weeks (Self-managed with weekly tip via text message)	Post-intervention at 6 and 12 weeks.	PCL-C 14-item Use, satisfaction, and perceived effectiveness questionnaire.	6 weeks = 8% 12 weeks = 0% *NS*	6 weeks: 90% read weekly tip text messages. 75% found it at least moderately helpful. 68% were mostly to very satisfied with the website. 12 weeks: 96% read weekly tip messages. 91% found it at least moderately helpful. 90% were mostly to very satisfied with the website.	13.6% = experienced confusion with the website. 18% = unsuccessfully wanted to use a smartphone instead of a computer.	NS PCL-C results not reported.
Tiet 2019	Primary care patients (veterans)	Smartphone ownership. Positive screen (≥3/4) on the PC-PTSD screener.	App^1^	16 weeks (Self-managed with 5–10 min biweekly telephone support)	Post-intervention at 16 weeks.	PCL-C 5-item helpfulness of the App and brief telephone support questionnaire.	0% *70%. = used MA 1-month post download. 97%. = used MA 2 months post download. 72%. = used MA 3 months post download. 80% = used the MA at least two to three times per week. 91% = found phone support somewhat/very helpful.* * 73.6% = said that phone support impacted both the frequency and consistency of MA use somewhat/very positively.*	91% = found phone support somewhat/very helpful. 73.6% = found phone support impacted frequency and consistency of MA use somewhat/very positively.	NS	Non sig. decrease (*p* = .30) in total PCL-C scores between baseline (*M* = 35.76; *SD* = 14.67) and post-intervention (*M* = 34.52; *SD* = 1.17) (Cohen's *d* = .30). Sig. decrease (*p* = 0.035) in the PCL-C re-experiencing subscale between baseline (*M* = 10.66; *SD* = 4.64) and post-intervention (*M* = 9.41; *SD* = 4.71) (Cohen's *d* = .71).
Pacella-LaBarbera 2020	In-patient motor vehicle crash (MVC) / motorcycle crash (MCC)	Within 24 h of an MVC/MCC (inclusive of pedestrian or bicycle crash) Musculoskeletal injury. Pain score = ≥4 (e.g. ‘On a scale of 0–10, how severe is your pain?’). English-speaking. Smartphone owner with the ability to download App. *MVC/MCC caused by a medical condition (e.g. syncope). Presence of: significant damage to subcutaneous tissue, specific nerve injury or neurological disease, moderate to* severe cognitive impairment secondary to head injury, *self-inflicted injury, benzodiazepine/ psychotropic medication/psychotherapy initiation while in an emergency department Not in active PTSD (pharmacologic treatment/ psychotherapy) treatment.*	App^1^	12 weeks (Self-managed with weekly text reminders)	Post-intervention at 4 and 12 weeks.	PCL-5 16 – item PTSS coping self-efficacy survey.	4 weeks = 17% 12 weeks = 11% *57.9% = used MA in the first week. 12.5% = used MA in the second week. 16.3% = used MA in the third week. 13.2% = used MA in the fourth week.* * 25.7% = used MA on Fridays. 38.5% = used MA between 9 pm – midnight.*	76% = at least moderately satisfied with MA. Participants found MA moderately to very helpful (*M* = 2.30; *SD* = 1.11). Non sig. change in PTSS coping SE from baseline (*M* = 70.12; *SD* = 1 9.26) to 30 day follow-up (*M* = 65.34; *SD* = 27.53).	67% = not enough time to use MA. 19% = felt MA was not needed.	Means were similar across conditions. No sig. effects of time/condition. Non sig. decrease (CI 95%* = *2.91–9.82) in total PCL-5 scores from 4 (*M* = 25.73; *SD* = 19.96) to 12 weeks (*M* = 19.33; *SD* = 18.97).^7^
Shakespear-Finch 2020	Ex-Serving Australian Defense Force members (veterans)	Ex-serving member of the Australian Defense Force	App^8^	Varied (Self-managed)	Once after app use.	Mobile Apps Rating Scale (4 subscales: Engagement, Functionality, Aesthetics, Information) rated from 1 = very poor to 5 = excellent; 4 subjective quality items. FGD Interviews.	0% *NS*	MA rated moderately high. MA rated moderately high (*M* = 3.87; *SD* = 0.46). Most endorsed features = ‘Information’ (*M* = 4.10; *SD* = 0.57) and ‘functionality’ (*M* = 4.09; *SD* = 0.58). Most found MA easily accessible. Generally found audios, stress monitoring, and psycho-educational information helpful.	Knowledge: Lack of familiarity with MA. Unclear usefulness of MA. MA Content: Outdated aesthetics and lack of customisation; Dense text; Potential to trigger trauma.	NS
van der Meer et 2020.	Health care professionals	≥18 years. Work related TE. Positive (≥1) on PC-PTSD for DSM-5 (PC-PTSD-5). Health care worker. Smart device (phone or tablet) owner. Dutch language proficiency.	App^9^	8 weeks (Self-managed)	Post-intervention at 8 and 12 weeks.	PCL-5 Perceived helpfulness of and satisfaction with SUPPORT Coach survey.	8 weeks = 23% 12 weeks = 8% *NS*	81.9% = found MA easy to use. 67.5 = perceived helpful to learn about PTSD symptoms. 59% = overall satisfied with MA.	NS	Non sig. between conditions effect size estimates (*p *= −.41) in total PCL-5 scores. Sig. decrease in total PCL-5 scores in both conditions from baseline to post-intervention. Int. group: (*p = -.35*) Control group: (*p *= .000).
Hensler 2022	Community	≥18 years. TE exposure within the past 2 years. PCL-5 ≥10. Resides in Sweden. Swedish verbal and written comprehension Smartphone owner. *Harmful living conditions (i.e. chronic TE exposure). Psychiatric co-morbidity: (1) current (past 6 months) alcohol or substance abuse disorder* *(2) severe suicidal ideation, (3) lifetime manic, hypomanic or psychotic episodes.* *Current/ pending psychotherapy. Medical treatment changes and/or current medication with counterindications (i.e. benzodiazepine).*	App^3^	12 weeks (Self-managed)	Post-intervention at 12 weeks.	PCL-5 15-item self-report PTSD Coach survey. Negative Effects Questionnaire.	12 weeks = 16% *NS*	72% = were moderately or very satisfied with the MA (*M* = 2.22; *SD* = 1.07). Perceived helpfulness = slightly to moderately helpful (*M* = 23.11; *SD* = 14.32).	20% = unfulfilled expectations on the MA. 18% = found MA unmotivating. 13% = psychological distress. 11% = found MA confusing.	Non sig. between conditions effect size estimates (Cohen's *d *= −.45, 95% CI −0.70 to −0.20) in total PCL-5 scores.
Miller-Graff 2021	Community	≥18 years. PCL-5 ≥33. Egyptian. Egyptian Arabic first language proficiency. *Severe mental health and/or psychotic symptoms.*	PCO^10^	6 weeks (Self-managed)	Post-intervention at 6 and 12 weeks.	PCL-5 Survey to evaluate the use of PCO.^10^	6 weeks = 21% 12 weeks = 28% *78% = used PCO-Arabic.* * 22% = used PCO-Arabic weekly. 1 h = average weekly usage.*	‘Notice Your Thoughts and Feelings’ tool most used (*M* = 2.67; *SD* = 0.46) and perceived most beneficial (*M* = 2.60; *SD* = 0.63).	Further exploration for cultural appropriateness/adaptation based on poorly rated tools: ‘Look Carefully at Your Thoughts, ‘Change Feelings by Changing Thoughts’, and ‘Improve Sleep Habits.’	Non sig. between conditions effect size estimates (Cohen's *d *= .14, 95% CI 0.82–0.53) in total PCL-5 scores. Int. Group Non sig. difference (Cohen's *d* = −.13, 95% CI −0.98–0.53), in total PCL-5 scores from baseline to post-intervention.
Ellis 2022	Community	Participants in the PCO arm of the Miller-Graff 2021 study.	PCO^10^	6 weeks (Self-managed)	Post-intervention at 6 and 12 weeks.	Semi-structured interviews.	6 weeks = 21% 12 weeks = 28% *NS*	53% = found the programme user-friendly and well organised. 53% = increased openness to other types of psychotherapy. 47% = found the programme acceptable and beneficial.^10^ 27% = programme strategies easily applicable in daily life. 20% = found the programme user friendly and relevant.	Main barrier reported is a lack of customisation. 73.3% = lack of human element. 33% = difficult to access due to the interface, website structure, and organisation of the programme. 53% = dissatisfied with programme content.	NS
Hallenbeck 2022	General population	iOS / Android App (Version 3.1) users.	App^1^	NS	1 year observation period.	PCL-5 Mobile analytics	207,001 total downloads. 90.92% = United States of America. 73.65% = iOS downloads. *72% = used MA at any point. 10% = used MA monthly. 0.69% = used MA 1-year post download. Users = used MA for approximately 18 min across 3 days. 87% = used MA first day post download.* * 8.53% = used MA 2 days post download.*	iOS users = sig. lower levels (*p* = <.001) of satisfaction.	NS	Sig. change (Cohen's *d* = −4.35, CI −4.77 to −3.92) from the 2.4% who completed PCL-5 twice.
Bröcker 2022a	Community	18 - 65 years. CAPS-5 ≥ 23. Conversational English proficiency. Able to attend weekly sessions at the university campus during office hours. Stable psychotropic medication use for ≥2 months pre-study enrolment. *Current (past 6 months) alcohol or substance abuse disorder.*	PCO	8 weeks (counsellor supported)	Mid-intervention at 4 weeks and post-intervention at 8 weeks.	CAPS-5 Field notes	4 weeks = 10% 8 weeks = 20% *NS*	Generally positive feedback (counsellor support and structured sessions).	Computer literacy was problematic.	57% = Sig. improvement in CAPS-5 scores in completers (RCI = <1.96). Int. Group: 2/3 completers improved significantly (RCI = <1.96) Control Group: 2/3 completers improved significantly (RCI = <1.96).
Bröcker 2022b	Community	18 - 65 years. CAPS-5 ≥ 23. Conversational English proficiency. Able to attend weekly sessions at the university campus during office hours. Stable psychotropic medication use for ≥2 months pre-study enrolment. *Current (past 6 months) alcohol or substance abuse disorder.*	App^1^	4 weeks (Self-managed and Counselor – supported)	Post-intervention at 4 weeks.	CAPS-5 Perceived helpfulness of the PTSD Coach MA survey. Field notes.	4 weeks = 20% *NS*	Generally, participants found the application helpful. Comparatively, the counsellor-supported grou*p* = found it more helpful (*p *= .04).	Problematic phone memory to download MA was reported once.	100% = Sig. improvement in CAPS-5 scores (RCI = <1.96) in completers. Counsellor Supported group: 2/5 completers improved significantly (RCI = <1.96) from extreme to mild severity. 2/5 completers improved significantly (RCI = <1.96) from severe to moderate severity. 1/5 completers improved significantly (RCI = <1.96) from extreme to moderate severity. Self-managed group: 2/4 completers improved significantly (RCI = <1.96) from severe to moderate severity. 1 / 4 completers improved significantly (RCI = <1.96) from extreme to severe severity.

Notes*:* ^1^Original PTSD Coach Mobile App version available from the app store; ^2^Non-public, research version of PTSD Coach Mobile App; ^3^Post-intervention is the first follow-up reported when there are more than one follow-up time points; ^4^12 and 16-week follow-up not reported on due to possible confounding factors described by authors; ^5^PTSD Coach Sweden; ^6^Health is Our Tradition: Balance and Harmony after Trauma (Fully adapted PTSD Coach Online); ^7^Changed score from 4 to 12 weeks reported, since post-traumatic stress symptoms keyed to index trauma not assessed at baseline. ^8^PTSD Coach Australia; ^9^SUPPORT Coach (the Netherlands); ^10^PTSD Coach Online – Arabic.

CAPS-5 = Clinician-Administered PTSD Scale for DSM-5.; CI = Confidence Interval; CS-PTSD – Clinician Supported-PTSD Coach; DSM-IV = Diagnostic and Statistical Manual of Mental Disorders, 4^th^ edition; DSM-V = Diagnostic and Statistical Manual of Mental Disorders, 5th edition; FGD = Focus Group Discussion; Int. = Intervention; *ITT* = Intention to Treat analysis; MA = Mobile Application; NA = Not Applicable; M = Mean; MA = Mobile Application; NS = Not Specified; PC-PTSD = Primary Care-PTSD Screen; PC-PTSD-5 = Primary Care-PTSD Screen for DSM-5; PCL-C = PTSD Checklist – Civilian version; PCL-S = PTSD Checklist-Specific; PTSD = Posttraumatic Stress Disorder; PTSS = Post Traumatic Stress Symptoms; RCI = Reliable Change Index; SD = Standard Deviation; Sig – Significant. SM-PTSD = Self-managed PTSD Coach.

Half of the primary studies (*n* = 8; 50%) required self-reported PTSS (i.e. PCL) for inclusion, with four (25%) using the Clinician-Administered PTSD Scale for DSM-5 (CAPS-5) (Weathers et al., 2013) to evaluate for the presence of both PTSS and PTSD.

Most primary studies (*n* = 13; 81.25%) evaluated PTSD Coach MA as the intervention with four studies evaluating a fully adapted version of the MA (Cernvall et al., 2018; Hensler et al., 2022; Shakespeare-Finch et al., 2020; van der Meer et al., 2020). In the studies that evaluated PCO (*n* = 3; 18.75%), two evaluated fully adapted versions (Hiratsuka et al., 2019; Miller-Graff et al., 2021). Most studies (*n* = 11; 68.75) evaluated a self-managed intervention, with two (12.5%) augmenting self-managed use with weekly tip messages/phone calls, and the remainder (*n* = 3) evaluating a supported (i.e. clinician/counsellor) intervention.

Intervention duration varied from three days to 16 weeks with the two multi-country studies not specifying intervention duration. Apart from the multi-country studies that included a one-year observation period, post-intervention follow-up time points for the remainder of the studies varied from immediately after PTSD Coach MA/PCO use to 24 weeks.

In the studies that reported outcome variables for changes in PTSS (*n* = 9), four used the PTSD Checklist for DSM-5 (PCL-5), while three used the PCL Civilian version (PCL-C), and one the PCL-Specific (PCL-S). The PCL-5 is a 17-item self-report PTSD symptom measure with internal consistency (Cronbach's α = .96) aligned with the DSM-5 (Bovin et al., 2016). Similarly, both the PCL-C and PCL-S are 17-item self-report PTSD symptom measures aligned with the DSM-IV. Both the PCL-C (Cronbach's α = .939) (Blanchard et al., 1996) and PCL-S (Cronbach's α = .91) (de Paula Lima et al., 2012) have good internal consistency based on original validation studies.

Two primary studies reported specifics on smart device ownership (Kuhn et al., 2014; Miner et al., 2016), while either ownership or access to a smart device can be deduced from study inclusion criteria from the remainder of the studies.

### 3.2. Feasibility and acceptability

For this review, feasibility and acceptability were determined by considering attrition rates, PTSD Coach MA/PCO usage, smart device ownership, and feedback provided by the trauma-exposed study participants about perceived satisfaction/benefits and barriers/concerns.

#### 3.2.1. Attrition and usage

In the primary RCT studies, attrition rates ranged from 10% to 23% at post-intervention follow-up (Bröcker et al., 2022; Hensler et al., 2022; Kuhn et al., 2017; Miller-Graff et al., 2021; Miner et al., 2016; Possemato et al., 2016; van der Meer et al., 2020). Attrition rates ranged from 0% to 8% at the post-intervention follow-up in the four pre-post primary studies (Cernvall et al., 2018; Hiratsuka et al., 2019; Kuhn et al., 2014; Tiet et al., 2019).

Owen et al. (2015) indicated that regular use of the MA decreased from 61.1% after the initial download, to 46% at one month, 28% at 3 months and 10.6% at approximately 12 months post-download. This decline in regular MA usage appeared to be influenced by the type (iOS vs. Android) of smart device operating system (OS) that was used, with Android users being less likely to use the MA at one month post download than iOS users (Owen et al., 2015). Hallenbeck et al. (2022) reported that while most downloads (73.7%) were from iOS users they showed significantly lower regular MA usage levels than Android users. Furthermore, they report that 72% of all users engaged with the MA at any point, with regular MA usage declining from 87% the first day post download to 10% and 0.69% one month and one year post download (Hallenbeck et al., 2022).

In contrast, Tiet et al. (2019) reported that 70% of users engaged with the MA one month post download, and 97% and 72% two and three months later, respectively. However, the influence of smart device OS type was not reported. The latter study as well as other primary study data suggest that both less study attrition rates and higher MA engagement are positively influenced by a type of support (i.e. telephonic/clinician support) (Bröcker et al., 2022; Hiratsuka et al., 2019; Pacella-LaBarbara et al., 2020; Possemato et al., 2016; Tiet et al., 2019).

#### 3.2.2. Satisfaction/benefits

Overall PTSD Coach MA users were moderately to very satisfied (Cernvall et al., 2018; Hensler et al., 2022; Kuhn et al., 2014, 2017; Pacella-LaBarbara et al., 2020; Possemato et al., 2016, 2017; van der Meer et al., 2020). According to one study, satisfaction was not correlated with age, but smart device ownership as opposed to non-ownership increased satisfaction (Kuhn et al., 2014). Augmenting MA use with support also increased satisfaction (Possemato et al., 2016, 2017); and satisfaction rates were generally high for PCO (Bröcker et al., 2022; Ellis et al., 2022; Hiratsuka et al., 2019).

Participants generally perceived the PTSD Coach MA as helpful (Bröcker et al., 2022; Hensler et al., 2022; Kuhn et al., 2014; Miner et al., 2016; Owen et al., 2015; Pacella-LaBarbara et al., 2020; Possemato et al., 2017; Shakespeare-Finch et al., 2020; van der Meer et al., 2020). This perception seems irrespective of the type of smart device OS (Kuhn et al., 2014), however, one study reported comparatively higher levels of positive perception in iOS than Android users (Owen et al., 2015). Again, augmenting MA use with support seemed to increase positive perception (Bröcker et al., 2022; Possemato et al., 2016, 2017; Tiet et al., 2019). Some helpful components highlighted were managing symptoms (Miner et al., 2016), learning about PTSD (Cernvall et al., 2018; van der Meer et al., 2020), information, and functionality (Shakespeare-Finch et al., 2020).

Notably, not all participants had a positive experience with the MA as, for example, was reported by 13.2% of the participants in the Miner et al. (2016) study. Perception of improved ability to cope with PTSD also did not increase post MA use (Kuhn et al., 2017; Pacella-LaBarbara et al., 2020).

Again, rates of perceived helpfulness were generally high for PCO (Bröcker et al., 2022; Ellis et al., 2022; Hiratsuka et al., 2019). Augmenting the PCO programme with support received positive feedback (Bröcker et al., 2022; Hiratsuka et al., 2019); user friendliness, organisation, and relevance of the programme were also noted (Ellis et al., 2022).

#### 3.2.3. Barriers/concerns

Smart device ownership was not a significant barrier to intervention feasibility (Bröcker et al., 2022; Cernvall et al., 2018; Hensler et al., 2022; Hiratsuka et al., 2019; Kuhn et al., 2014, 2017; Miller-Graff et al., 2021; Miner et al., 2016; Pacella-LaBarbara et al., 2020; Possemato et al., 2016; Shakespeare-Finch et al., 2020; Tiet et al., 2019; van der Meer et al., 2020). However, one study mentioned that owners reported more PTSS improvement than non-owners (Miner et al., 2016) while another study mentioned phone memory hindered initial MA download (Bröcker et al., 2022).

Some participants found the structure (Cernvall et al., 2018; Hensler et al., 2022; Owen et al., 2015) and content (Shakespeare-Finch et al., 2020) of the MA problematic. Difficulties and confusion with the PCO website were also reported (Ellis et al., 2022; Hiratsuka et al., 2019). This links to the barrier of the inability to customise some features of the MA (Kuhn et al., 2014; Owen et al., 2015; Shakespeare-Finch et al., 2020), which was also noted as a barrier to PCO use (Ellis & Miller-Graff, 2021).

General technical glitches were another barrier experienced by MA users (Cernvall et al., 2018; Owen et al., 2015). The technical problems appear to be influenced by the type of smartphone device OS used, with Android users reporting significantly more technical problems than iOS users (Owen et al., 2015). For one user, the technical difficulties resulted in increased psychological distress (Owen et al., 2015). Other participants also mentioned potential psychological distress as a barrier (Hensler et al., 2022; Shakespeare-Finch et al., 2020). Concerning PCO, computer literacy (Bröcker et al., 2022) and the inability to use the website on a smartphone (Hiratsuka et al., 2019) hindered use.

Other barriers to feasibility and acceptability of the MA included differences in expectation (Cernvall et al., 2018; Hensler et al., 2022), the perceived need for MA use (Pacella-LaBarbara et al., 2020; Shakespeare-Finch et al., 2020), and insufficient time availability (Pacella-LaBarbara et al., 2020). Dissatisfaction with the content was noted by some MA users (Hensler et al., 2022) as well as by some PCO participants (Ellis et al., 2022).

Lastly, a few MA participants suggested integrating other support sources rather than using the MA as a stand-alone intervention (Cernvall et al., 2018; Owen et al., 2015; Shakespeare-Finch et al., 2020). Linked hereto is that most PCO participants noted the lack of a human element as a barrier (Ellis et al., 2022).

#### 3.2.4. Effectiveness

Effectiveness was determined by considering a reduction in PTSS evaluated with validated subjective or diagnostic measures (i.e. PCL / CAPS-5). Where the included studies had more than one follow-up point, data from baseline to post-intervention was used. For the meta-analysis, we included the six PTSD Coach MA RCTs that reported on PCL outcome data (Hensler et al., 2022; Kuhn et al., 2017; Miner et al., 2016. Pacella-LaBarbara et al., 2020; Possemato et al., 2016; van der Meer et al., 2020). Due to variability in study design and outcome measures (PCL-C, PCL-S and PCL-5) a random effects model and standardised mean difference (SMD) were used to calculate effect sizes. Some studies reported a significant decrease in total PCL scores between baseline and post-intervention in the intervention group but not in the waitlist control groups (Kuhn et al., 2017; Miner et al., 2016), while others reported a significant decrease in both study groups in the respective RCTs, namely self-managed PTSD Coach versus clinician supported PTSD Coach, and access to versus no access to SUPPORT Coach (Possemato et al., 2016; van der Meer et al., 2020). However, the overall greater decrease in symptom severity in the intervention group compared to the comparison group (pooled effect size) was not significant, SMD =  –0.19 (95% CI −0.41 to −0.03, *p* = .09). Heterogeneity was not significant (*p* = .14; I^2 ^= 40%).

The larger (*N* = 87) PCO RCT with treatment or waitlist control groups reported a non-significant decrease in total PCL scores from baseline and post-intervention between conditions (Cohen's *d* = – 0.14) (Miller-Graff et al., 2021).

Another small (*N* = 10) PTSD Coach MA RCT reported a significant decrease in total CAPS-5 score (Reliable change index = < 1.96) in all participants from baseline to post-intervention in the counsellor-supported arm, and in two participants in the self-managed arm (Bröcker et al., 2022).

Pre to post-intervention PCL data also showed a non-significant decrease in total PCL scores from baseline to post-intervention (Cernvall et al., 2018; Tiet et al., 2019), however of interest is the reported significant decrease in the PCL re-experiencing subscale score (*p* = .035) (Tiet et al., 2019). Although outside the scope of this review, it is worth noting that some studies (Cernvall et al., 2018; Hensler et al., 2022; Kuhn et al., 2017; Miller-Graff et al., 2021; Possemato et al., 2016; Tiet et al., 2019) reported on the intervention effects on depression as a secondary outcome. The potential broader benefit of ‘PTSD Coach’ on depressive symptoms as a secondary outcome was supported by some studies (Hensler et al., 2022; Kuhn et al., 2017; Possemato et al., 2016; Tiet et al., 2019), but not by others (Cernvall et al., 2018; Miller-Graff et al., 2021).

#### 3.2.5. Quality of included papers

**Figure 3.**
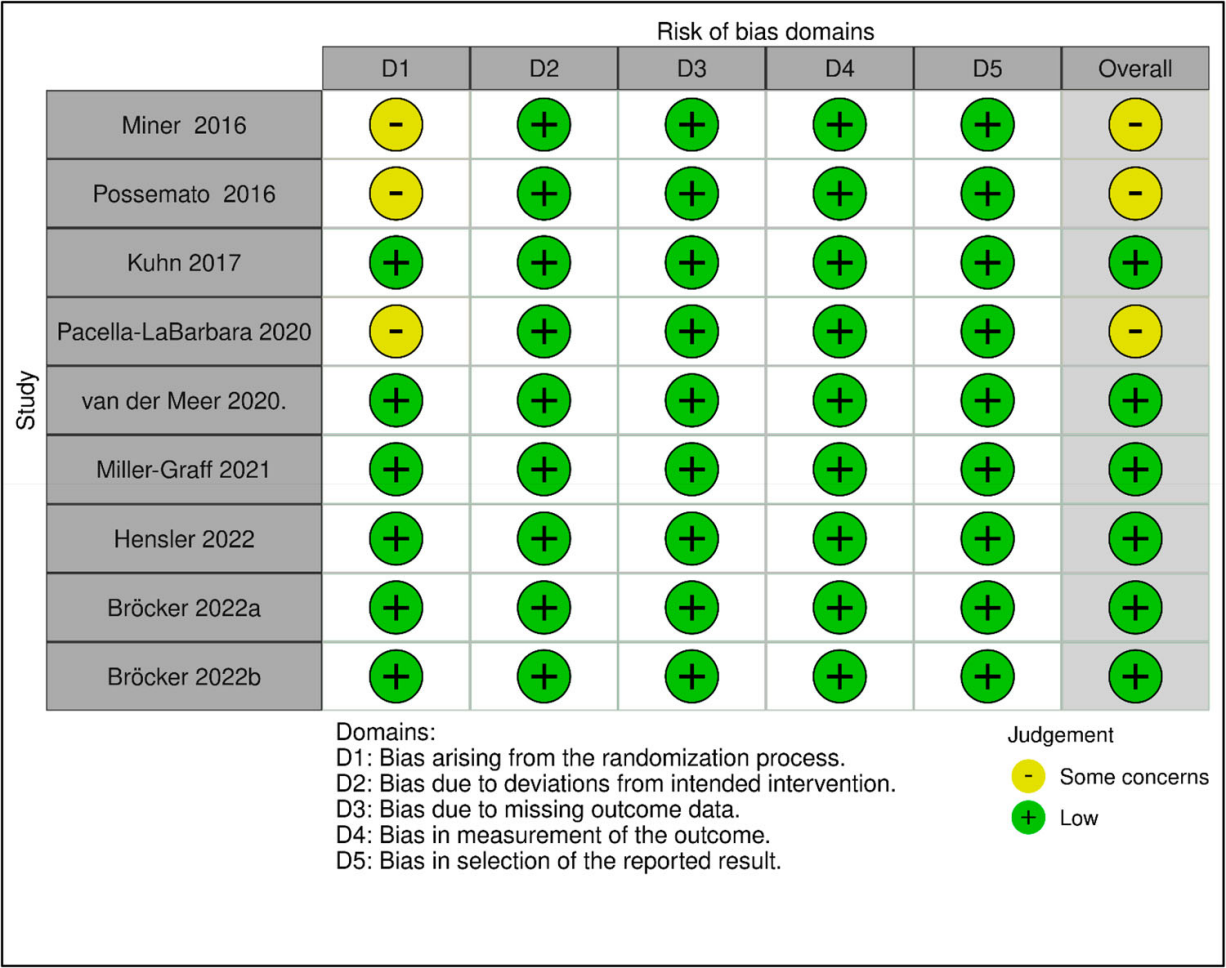
ROB-2 Quality appraisal of included articles. Notes. Bröcker’s paper consisted of two individual randomised controlled trial pilots and each pilot was assessed independently.

The results of the MMAT and ROB-2 are presented in Table 3 and Figure 3, respectively. In general, the quality of the manuscripts was deemed acceptable based on the different study designs. The main concerns were a lack of sufficient information concerning allocation concealment (*n = *3) and blinding to the intervention by outcome assessors (*n = *6), however, since the outcomes were self-reported the outcome data was not influenced; insufficient information on baseline group differences (*n* = 2); and small sample sizes (*n = *3) that may have affected representativeness. Reviewers queried a discrepancy found between the reported frequencies by ethnicity in Kuhn et al. (2017) where the reported numbers exceeded the number of enrolled participants (Table 1, pg. 270). The quality assessment of this study was not adversely affected since the author clarified that participants self-identified with more than one ethnic category.

**Table 3.** MMAT Quality appraisal of included articles.

MMAT Qualitative quality appraisal						
Study	1. Is the qualitative approach appropriate to answer the research question?	2. Are the qualitative data collection methods adequate to address the research question?	3. Are the findings adequately derived from the data?	4. Is the interpretation of results sufficiently substantiated by data?	5. Is there coherence between qualitative data sources, collection, analysis, and interpretation?	Final Rating
Ellis 2022	Yes	Yes	Yes	Yes	Yes	5

MMAT Quantitative RCT quality appraisal						
Study	1. Is randomisation appropriately performed?	2. Are the groups comparable at baseline?	3. Are there complete outcome data?	4. Are outcome assessors blinded to the intervention provided?	5. Did the participants adhere to the assigned intervention?	Final Rating
Miner 2016	No^1^	Yes	Yes	No^2^	Yes	5^3^
Possemato 2016	No^1^	Yes	Yes	Yes	Yes	5^3^
Kuhn 2017	Yes	Yes	Yes	No^2^	Yes	5
Pacella-LaBarbara 2020	No^1^	Yes	Yes	No^2^	Yes	4
van der Meer 2020.	Yes	Yes	Yes	No^2^	Yes	5^4^
Miller-Graff 2021	Yes	No^5^	Yes	No^2^	Yes	4
Hensler 2022	Yes	Yes	Yes	No^2^	Yes	5^4^
Bröcker 2022a	Yes	No^3^	Yes	Yes	Yes	4
Bröcker 2022b	Yes	No^3^	Yes	Yes	Yes	4

MMAT Quantitative non-RCT quality appraisal						
Study	1. Are the participants representative of the target population?	2. Are measurements appropriate regarding both the outcome and intervention (or exposure)?	3. Are there complete outcome data?	4. Are the confounders accounted for in the design and analysis?	5. During the study period, is the intervention administered (or exposure occurred) as intended?	Final Rating
Owen 2015	Yes	Yes	Yes	Yes	Yes	5
Cernvall 2018	Unsure^6^	Yes	Yes	Yes	Yes	4
Hiratsuka 2019	Unsure^6^	Yes	Yes	Yes	Yes	4
Tiet 2019	Unsure^6^	Yes	Yes	Yes	Yes	4
Hallenbeck 2022	Yes	Yes	Yes	Yes	Yes	5

MMAT Mixed Methods quality appraisal						
Study	1. Is there an adequate rationale for using a mixed methods design to address the research question?	2. Are the different components of the study effectively integrated to answer the research question?	3. Are outputs of the integration of qualitative and quantitative components adequately interpreted?	4. Are divergences and inconsistencies between quantitative and qualitative results adequately addressed?	5. Do the different components of the study adhere to the quality criteria of each tradition of the methods involved?	Final Rating
Kuhn 2014	Yes	Yes	Yes	Yes	Yes	5
Possemato 2017	Yes	Yes	Yes	Yes	Yes	5
Shakespear-Finch 2020.	Yes	Yes	Yes	Yes	Yes	5

Notes*.* Bröcker paper consisted of two pilot randomised controlled trials (a = Pilot 1; b = Pilot 2) and each pilot was assessed independently; ^1^No information regarding allocation concealment was provided, however, due to self-reported outcome data this likely did not result in bias; ^2^Outcome assessors were not blinded to the intervention provided, however, since the outcomes were self-reported the outcome data was not influenced; ^3^Taking into account explanations^1,2^; ^4^Taking into account explanation^2^; ^5^No specific information regarding the presence/absence of baseline imbalances provided; ^6^small community sample that may affect the sample representativeness as mentioned in study limitations.

1 = 20% quality criteria met; 2 = 40% quality criteria met; 3 = 60% quality criteria met; 4 = 80% quality criteria met; 5 = 100% quality criteria met

## 4. Discussion

This systematic review provided a synthesis of the feasibility, acceptability, and effectiveness of PTSD Coach MA and PCO data in trauma-exposed individuals and highlighted intervention challenges and recommendations for future research. Seventeen manuscripts reporting on 16 primary studies were included in the review. Findings support the feasibility and acceptability of ‘PTSD Coach’ in trauma-exposed individuals, however evidence for its effectiveness is currently limited.

Generally, users were satisfied with both ‘PTSD Coach’ platforms, however, the type of smart device OS (i.e. iOS or Android) affected MA use and satisfaction with a suggestion to develop OS specific versions of the MA (Owen et al., 2015). Perceived helpfulness for both ‘PTSD Coach’ platforms was generally high, with type of smart device OS use again playing a role. Differences in experience of and preference for either iOS or Android are frequently debated among users and developers (Győrödi et al., 2017; Kaur Ubhi et al., 2017; Wang & Godfrey, 2013)

Like other PTSD intervention studies, this review found that most of the studies used the PCL to monitor intervention effects on PTSS and one study used the CAPS-5 (Steubl et al., 2021). Some PTSD Coach MA studies reported a significant reduction in PTSS in the intervention group (Kuhn et al., 2018; Miner et al., 2016). However, as seen in Figure 3, the pooled effect size was not significant. Tiet et al. (2019) was the only study that reported intervention effects on PCL subscales in which they found a significant decrease in the re-experiencing subscale. This level of subscale analysis can inform which PTSD symptom clusters are more sensitive to ‘PTSD Coach’, and possibly aid in either a more targeted intervention approach or intervention refinement.

Most studies were conducted in HICs. This may be due to ‘PTSD Coach’ originating in a HIC, and comparatively higher internet access and smart device ownership compared to LMICs (Campbell et al., 2015; Kuhn et al., 2018; Stork et al., 2013). Additionally, significantly higher treatment-seeking behaviour (53.5%) in HICs compared to LMICs (22.8%) likely contributed (Koenen et al., 2017). Aside from the ‘PTSD Coach’ platforms, other internet – and mobile-based interventions for PTSD were also mostly conducted in HICs (Steubl et al., 2021).

The over-representation of females in the 14 studies that reported on sex is interesting since ‘PTSD Coach’ was originally developed for veterans (mostly men) (Harrington et al., 2019). However, the over-representation of females agrees with general PTSD research indicating a higher prevalence of PTSD reported by women, who are also more likely to seek treatment (Koenen et al., 2017; Olff, 2017). Furthermore, the lack of specification of ethnicity in the studies is noteworthy. It is important to expand this research to other groups considering ‘PTSD Coach’ research has suggested a difference in treatment response rates in subgroup analyses (Pacella-LaBarbara et al., 2020), further highlighting the importance of stratifying by sex/ethnicity.

While the quality of the included papers can generally be deemed acceptable, future studies should be more rigorous in terms of reporting on allocation concealment and blinding of outcome assessors, providing sufficient baseline information, and including larger sample sizes.

In conclusion, most of the studies evaluated a self-managed ‘PTSD Coach’ intervention with a select few evaluating an augmented (i.e. weekly tip messages/phone calls and clinician/counsellor) intervention delivery. The studies that evaluated a supported ‘PTSD Coach’ intervention generally seemed to have less attrition, higher engagement, and increased perceived helpfulness and satisfaction (Bröcker et al., 2022; Hiratsuka et al., 2019; Possemato et al., 2016, 2017; Tiet et al., 2019).

### 4.1. Strengths and limitations

To our knowledge, this is the first review to combine quantitative and qualitative studies on both ‘PTSD Coach’ platforms, resulting in a richer picture of the currently available research. However, only including peer-review published research may be a limitation, as we may have missed useful information included in unpublished research. Additionally, the relatively small number of eligible RCTs and small sample sizes limits the evidence on whether PTSD Coach is an effective intervention for reducing PTSS. Nonetheless, this is the first ‘PTSD Coach’ review that includes a meta-analysis indicating an increase in both general research advances in this area and specifically in effectiveness data as was a need suggested by previous reviews (Gould et al., 2019; Kuhn et al., 2018).

### 4.2. Future research

These findings suggest that more research is needed in LMICs, particularly that which evaluates supported ‘PTSD Coach’ interventions in larger and more diverse (i.e. sex and ethnicity) samples. Additionally, objective monitoring of PTSS (i.e. blinded monitoring with diagnostic measures such as the CAPS-5) in conjunction with self-report measures is needed. Although many studies have been conducted since the last review (Kuhn et al., 2018) more research is needed to increase evidence for the effectiveness of ‘PTSD Coach’ as an intervention for PTSS. A focus on improvements related to PTSD symptom clusters is also recommended to further inform intervention effectiveness and/or improvements. The evaluation of PTSD Coach intervention effects on co-morbid disorders such as anxiety and depression may also prove insightful. Other authors have suggested that ‘PTSD Coach’ be evaluated as an augmented intervention (i.e. supported) (Miller-Graff et al., 2021; Miner et al., 2016) or augmentative intervention (i.e. alongside psychotherapy) (van der Meer et al., 2020) as part of future research agendas.

In sum, findings support the feasibility and acceptability of ‘PTSD Coach’ in trauma-exposed individuals. However, evidence on the effectiveness on PTSS remains limited; and especially in LMIC where there is limited access to these interventions, and/or stigma around accessing mental health services more research is needed.

## Supplementary Material

Supplemental Material

Supplemental Material

Supplemental Material

Supplemental Material

## Data Availability

The data that support the findings reported in this review can be made available from the corresponding author (E. Bröcker) upon reasonable request.
