# Supplementary material for: Feasibility, acceptability, and effectiveness of web-based and mobile PTSD Coach: a systematic review and meta-analysis
Source: Eur J Psychotraumatol. 2023 May 25;14(2):2209469. doi: 10.1080/20008066.2023.2209469 (PMC10215014; doi:10.1080/20008066.2023.2209469)
Supplement: Supplemental Material [file ZEPT_A_2209469_SM9689.pdf]

Eligibility form

Record ID	______________________
Study ID:<br>(surname, first name of the main author, and publication year - e.g. Van der Meer, 2011)	______________________
Reviewer initials	______________________
Date form completed	______________________
STUDY DESIGN	
1. The study is a quantitative, qualitative, or mixed methods primary research study reporting on the feasibility, acceptability, and/or effectiveness of PTSD Coach (web-based or mobile application) as treatment or prevention?	◯ Yes<br>◯ No<br>◯ Unclear
Notes	______________________
PARTICIPANTS / POPULATION	
2. The study is in participants of all ages that focus on PTSD Coach (web-based or mobile application) ?	◯ Yes<br>◯ No<br>◯ Unclear
Notes	______________________
3. The study includes trauma-exposed individuals?<br>(fulflling DSM/ICD criteria)	◯ yes<br>◯ No<br>◯ Unclear
Notes	______________________
INTERVENTION	
4. The study evaluates PTSD Coach (web-based or mobile application) as an intervention?	◯ Yes<br>◯ No<br>◯ Unclear
Notes	______________________
COMPARATOR(S)/CONSTROL ARM(S)	

5. The study compares PTSD Coach (web-based or mobile application) to any comparator (i.e. treatment as usual / wait list control etc) ?	◯ Yes<br>◯ No<br>◯ Unclear
Notes	__________________________________
6. The study has no comparator (i.e qualitative research investigating user experience/pre to post)?	◯ Yes<br>◯ No<br>◯ Unclear
REVIEWER DECISION	
7. Include in this review?<br>(Answers 1 - 4 = "Yes" and 5 or 6 = "Yes")	◯ Yes<br>◯ No<br>◯ Unclear/Uncertain
Notes	__________________________________
8. Is additional information required from the study authors before a final assessment/decision can be made?	◯ Yes<br>◯ No
Notes	__________________________________
9. If yes to the above, what information is required before a decision can be made?	__________________________________
Notes	__________________________________
COMPLETE AFTER BOTH PRIMARY REVIEWERS HAVE INDEPENDENTLY COMPLETED THE ELIGIBILITY REVIEW	
10. Do both reviewers agree?	◯ Yes<br>◯ No
Notes	__________________________________
11. In the even that there was disagreement, was this resolved through discussion?	◯ Not applicable<br>◯ Yes<br>◯ No
Notes	__________________________________
12. In the event that disagreement was not resolved through discussion, what was the secondary reviewer's decision?	◯ Not applicable<br>◯ Include<br>◯ Exclude
Notes	__________________________________

13. In summary, reason for exclusion is (if applicable) ? ______________________

Notes ______________________
