# Supplementary material for: Feasibility, acceptability, and effectiveness of web-based and mobile PTSD Coach: a systematic review and meta-analysis
Source: Eur J Psychotraumatol. 2023 May 25;14(2):2209469. doi: 10.1080/20008066.2023.2209469 (PMC10215014; doi:10.1080/20008066.2023.2209469)
Supplement: Supplemental Material [file ZEPT_A_2209469_SM9688.pdf]

Data extraction form

Record ID ______________________

Study ID (surname of first author and year first full report of study was published e.g. Smith 2001) ______________________

Date form completed (dd/mm/yyyy) ______________________

Name/ID of person extracting data ______________________

Notes ______________________

CHARACTERISTICS OF INCLUDED STUDIES

1. Author(s)
   (>5 authors write first author et al.). ______________________

2. Year of publication ______________________

3. Journal results was published in ______________________

3. Page numbers in journal ______________________

4. DOI ______________________

5. Author contact details (e.g. e-mail) ______________________

Notes ______________________

6. Publication type
   - ◯ Full report
   - ◯ Brief report
   - ◯ Letter
   - ◯ Abstract
   - ◯ Conference report
   - ◯ Book chapter
   - ◯ Other
   - ◯ Not stated/unclear

7. Study funding details (if no funding - N/A) ______________________

8. Conflict of interests (declared or noticed)
   - ◯ Yes
   - ◯ No

8(a). If Yes to the above - explain/describe ________________________________

General notes for this section ________________________________

STUDY POPULATION AND SETTING

1. Country where study was conducted (include city if provided e.g. Amsterdam, The Netherlands) ________________________________

Notes ________________________________

2. Study population description (from where were participants were drawn) ________________________________

Notes ________________________________

3. Study setting (can include location - e.g. clinic; social context - e.g. resource constrained) ________________________________

Notes ________________________________

4. Inclusion criteria ________________________________

Notes ________________________________

5. Exclusion criteria ________________________________

Notes ________________________________

6. Recruitment methods ________________________________

Notes ________________________________

7. Informed obtained

- ◯ YES
- ◯ NO
- ◯ UNCLEAR

Notes ________________________________

8. Was Ethics approval needed?

- ◯ YES
- ◯ NO
- ◯ UNCLEAR

8a. If the yes to above, was ethics approval obtained?	◯ YES<br>◯ NO<br>◯ UNCLEAR
Notes	__________________________________
General notes for this section	__________________________________
STUDY METHODS	
1. Aim(s) and objective(s) of study (e.g. feasibility, acceptability, efficacy)	__________________________________
Notes	__________________________________
2. Study design (e.g. parallel, crossover, non-RCT, mixed)	☐ RCT Individually-randomized parallel-group<br>☐ RCT Cluster-randomized parallel-group<br>☐ RCT Individually randomized cross-over (or other matched)<br>☐ NON-RCT Pre to Post<br>☐ QUALITATIVE<br>☐ MIXED METHODS<br>☐ OTHER
Notes	__________________________________
2a. If yes to randomisation above - how was this done (e.g. computerised/manually).	__________________________________
2b. If yes to randomisation above, what method was used (e.g. stratified, 1:1 ratio)	__________________________________
Notes	__________________________________
3. Start and end date of study	__________________________________
3a. If the actual start and end date of the study is not provided - the duration of the entire study	__________________________________
Notes	__________________________________
5. Duration of participation (from recruitment/enrolled to last follow-up)	__________________________________
Notes	__________________________________

6. Symptom level required for participation

☐ TRAUMA EXPOSED (Clinical diagnosis of PTSD made by diagnostic measure)
☐ TRAUMA EXPOSED (Significant PTSD symptoms based on the subjective report(s))
☐ UNCLEAR

Notes

____

7. Method(s) (measure(s)) used to determine trauma exposure (e.g Diagnostic assessment, subjective measures)

____

Notes

____

8. Method(s) (measure(s)) used to determine PTSD diagnostic status and/or symptoms

____

Notes

____

9. Was co-morbidity allowed?

◯ YES
◯ NO
◯ UNCLEAR

Notes

____

9a. Method(s) (measure(s)) used to assess for the presence and severity of comorbidity if applicable

____

Notes

____

General notes for this section

____

STUDY PARITICIPANTS

1. Study sample
(Total no. randomised/total no. at start of study for non-RCTs)

____

1a. Clusters - if applicable, no., type, no. people per cluster)

____

Notes for above (can include quotes from the text, location in the text and reviewer explanations)

____

2. Baseline imbalances identified/present/addressed (for non-RCTs write N/A)

◯ YES
◯ NO
◯ UNCLEAR

2a. If yes/unclear to above, provide further information

____

Notes for above (can include quotes from the text, location in the text and reviewer explanations)	______________________
3. Withdrawals and/or exclusions made during study procedures (either by the research staff of participants) (e.g adverse events, concerns etc).	◯ YES<br>◯ NO<br>◯ UNCLEAR
3a. If yes to the above, provide further information	◯ YES<br>◯ NO<br>◯ UNCLEAR
Notes for above (can include quotes from the text, location in the text and reviewer explanations)	______________________
4. Age<br>(e.g. 24 to 64 years (M=:46.3; SD=13.82).	______________________
Notes for above (can include quotes from the text, location in the text and reviewer explanations)	______________________
5. Sex	______________________
Notes for above (can include quotes from the text, location in the text and reviewer explanations)	______________________
6. Race/Ethnicity	______________________
Notes for above (can include quotes from the text, location in the text and reviewer explanations)	______________________
7. Severity of illness (PTSD mild, moderate, severe, subthreshold, trauma exposed)	______________________
Notes for above (can include quotes from the text, location in the text and reviewer explanations)	______________________
8. Co-morbidities	______________________
Notes for above (can include quotes from the text, location in the text and reviewer explanations)	______________________
9. Other treatment received<br>(additional to study intervention)	______________________
Notes for above (can include quotes from the text, location in the text and reviewer explanations)	______________________
10. Other relevant sociodemographics	______________________
Notes for above (can include quotes from the text, location in the text and reviewer explanations)	______________________

General notes for this section ____________________

INTERVENTION(S)

1. What PTSD Coach platform was evaluated
- ☐ PTSD Coach Mobile Application
- ☐ PTSD Coach Online Web-based
- ☐ Both

Notes for above (can include quotes from the text, location in the text and reviewer explanations) ____________________

2. Number of study groups/arms ____________________

Notes for above (can include quotes from the text, location in the text and reviewer explanations) ____________________

3. Description of intervention groups as noted in manuscript - e.g. Self Managed PTSD Coach (SM PTSD Coach) enhanced treatment as usual. For non-RCT studies, provide the pre-intervention/baseline group description). ____________________

3a. Number allocated to each group (e.g. PTSD Coach = 80, eTAU= 80 / PTSD Coach = 80). ____________________

Notes for above (can include quotes from the text, location in the text and reviewer explanations) ____________________

4. Duration of intervention/treatment/participation period ____________________

Notes for above (can include quotes from the text, location in the text and reviewer explanations) ____________________

5. Timing (e.g. frequency, duration of each visit/contact) ____________________

Notes for above (can include quotes from the text, location in the text and reviewer explanations) ____________________

6. Delivery (e.g. in person, virtual, telephonic) ____________________

Notes for above (can include quotes from the text, location in the text and reviewer explanations) ____________________

7. Compliance (Integrity/fidelity of intervention delivery) (Integrity/fidelity of intervention delivery for participants) if applicable ____________________

Notes for above (can include quotes from the text, location in the text and reviewer explanations) ____________________

8. Providers/people involved in intervention (e.g. profession, training etc.)	__________________________________
Notes for above (can include quotes from the text, location in the text and reviewer explanations)	__________________________________
9. Resource requirements to replicate intervention (e.g. staff numbers, costs / technology)	__________________________________
Notes for above (can include quotes from the text, location in the text and reviewer explanations)	__________________________________
10. Any other information relevant to intervention	__________________________________
General notes for this section	__________________________________

QUANTITATIVE OUTCOME(S)

1. Outcome name(s) reported on at follow-ups (e.g (PCL-5; DASS-21; CAPS-5)	__________________________________
2. Outcome definition (subjective report / diagnostics / qualitative) (e.g PCL-5 = Subj report; CAPS-5 = Diagnostic)	__________________________________
Notes for above (can include quotes from the text, location in the text and reviewer explanations)	__________________________________
3. Is the outcome measure(s) validated?	◯ YES<br>◯ NO<br>◯ UNCLEAR
Notes for above (can include quotes from the text, location in the text and reviewer explanations)	__________________________________
4. Time points that the outcome(s) was assessed (e.g. Baseline, 4 weeks etc)	__________________________________
Notes for above (can include quotes from the text, location in the text and reviewer explanations)	__________________________________
5. Time points outcome(s) reported on (e.g pre- and post intervention at 8 weeks)	__________________________________
Notes for above (can include quotes from the text, location in the text and reviewer explanations)	__________________________________
6. Person measuring/assessing or reporting outcome(s)	__________________________________
Notes for above (can include quotes from the text, location in the text and reviewer explanations)	__________________________________

General notes

____

QUANTITATIVE RESULTS

1. Statistical methods used for analysis

____

1a. Appropriateness of these methods

- ◯ APPROPRIATE
- ◯ NOT APPROPRIATE
- ◯ UNCLEAR/UNSURE

Notes for above (can include quotes from the text, location in the text and reviewer explanations)

____

1b. Confounding factors/ effect modifiers accounted for in the analyses

- ◯ YES
- ◯ NO
- ◯ UNCLEAR

Notes for above (can include quotes from the text, location in the text and reviewer explanations)

____

1c. Was reanalysis required?
(specify why if yes)

- ◯ YES
- ◯ NO
- ◯ UNCLEAR

Notes for above (can include quotes from the text, location in the text and reviewer explanations)

____

2. N per group(s) at follow-up
(e.g.PTSD Coach = 60 and etau = 50)
(if more than one time follow-up timepoint extract for each time point)

____

Notes for above (can include quotes from the text, location in the text and reviewer explanations)

4. Reason(s) provided for attrition (lost to follow-up / withdraw etc).

____

Notes for above (can include quotes from the text, location in the text and reviewer explanations)

3. Attrition rate(s) reported (either percentage reported or self calculated)

____

Notes for above (can include quotes from the text, location in the text and reviewer explanations)

5. Reported data M/SD (e.g. (PTSD Coach-CS: M=3.4; SD=0.55 and PTSD Coach-SM: M=2.33; SD=0.58.)

____

5a. Note if other variance/results such as effect sizes are provided (e.g. Effect sizes provided in Table 2 on page 5).

____

Notes for above (can include quotes from the text, location in the text and reviewer explanations)	
6. Any other relevant quantitative data / comments / notes	__________________
Notes for above (can include quotes from the text, location in the text and reviewer explanations)	
QUALITATIVE OUTCOME(S)	__________________
1. How was qualitative data collected (structured or unstructured interviews, how many interviews, length of interviews, individuals or group interviews, face to face or telephonic interviews)(all information provided regarding of collecting this data	__________________
2. How many interviews / data sources (e.g. 10 interviews) were aimed to be collected/started with	__________________
3. Time point(s) of collection	__________________
4. Person(s) collecting outcome(s)	__________________
5. Any other relevant qualitative data notes	__________________
QUALITATIVE RESULTS	__________________
1. Statistical methods used for analysis	__________________
1a. Appropriateness of these methods	◯ APPROPRIATE<br>◯ NOT APPROPRIATE<br>◯ UNCLEAR/UNSURE
Notes for above (can include quotes from the text, location in the text and reviewer explanations)	__________________
1b. Confounding factors/ effect modifiers accounted for in the analyses	◯ YES<br>◯ NO<br>◯ UNCLEAR
Notes for above (can include quotes from the text, location in the text and reviewer explanations)	__________________
1c. Was reanalysis required? (specify why if yes)	◯ YES<br>◯ NO<br>◯ UNCLEAR
Notes for above (can include quotes from the text, location in the text and reviewer explanations)	__________________

2. How many data sources (e.g. interviews) analysed	______________________
3. Reasons for discrepancy between aimed number of data sources (e.g. interviews) and actual analysed	______________________
4. Author derived themes (all information documented in manuscript)<br>(1. ....)<br>(2. ....)	______________________
6. Any other relevant quantitative data / comments / notes	______________________

APPLICABILITY

1. Have important populations been excluded from the study?	◯ YES<br>◯ NO<br>◯ UNCLEAR
Notes for above (can include quotes from the text, location in the text and reviewer explanations)	______________________
2. Does the study directly address the research question?	◯ YES<br>◯ NO<br>◯ UNCLEAR
Notes for above (can include quotes from the text, location in the text and reviewer explanations)	______________________

OTHER INFORMATION

1. Key conclusions of study authors	______________________
2. Study limitations	______________________
3. Recommendations for future studies	______________________
4. Correspondence required for further study results information	◯ YES<br>◯ NO<br>◯ UNCLEAR
Notes for above (can include quotes from the text, location in the text and reviewer explanations)	______________________
5.Further study information requested (from whom, what and when)	
Notes for above (can include quotes from the text, location in the text and reviewer explanations)	______________________
6. Correspondence received (from whom, what and when)	______________________

Notes for above (can include quotes from the text, location in the text and reviewer explanations) ________________________________
